# Phenolic Profile and In Vitro Anti‐Inflammatory Activities of *Salvia officinalis* L. Hydrodistillation Wastewater

**DOI:** 10.1002/cbdv.202502271

**Published:** 2026-01-08

**Authors:** Maria Sofia Molonia, Federica Lina Salamone, Francesco Cimino, Manuela D'Arrigo, Mariateresa Cristani, Luana Pulvirenti, Antonella Saija, Antonio Speciale, Edoardo Napoli

**Affiliations:** ^1^ Department of Chemical, Biological, Pharmaceutical and Environmental Sciences University of Messina Messina Italy; ^2^ “Prof. Antonio Imbesi” Foundation University of Messina Messina Italy; ^3^ Institute of Biomolecular Chemistry – National Research Council (ICB‐CNR) Catania Italy

**Keywords:** *E. coli*, flavonoids, hydrodistillation wastewater, hydroxycinnamic acids, intestinal inflammation, *Salvia officinalis* L. essential oil

## Abstract

*Salvia officinalis* L. essential oil production process generates an exhausted residue of vegetable biomass, returned by hydrodistillation, along with a variable amount of wastewater enriched in water‐soluble compounds of high added value and biological activity. The aim of this study was to investigate the in vitro anti‐inflammatory effects of *S. officinalis* L. hydrodistillation wastewater using murine macrophage Raw 264.7 cells stimulated with *Escherichia coli* lipopolysaccharide (LPS) and human intestinal epithelial Caco‐2 cells exposed to *E. coli*. Polyphenolic compounds in wastewater were identified by high‐performance liquid chromatography‐mass spectrometry analysis, confirming the presence of organic acids (particularly hydroxycinnamic acids), flavones, and flavonols. This phytocomplex protected Raw 264.7 from *E. coli* LPS‐induced inflammation by reducing nuclear factor kappa B nuclear translocation and its transcriptional activity (interleukin [IL]‐6, IL‐8, and tumor necrosis factor‐alpha messenger RNA levels). Additionally, the wastewater reduced cyclooxygenase‐2 protein expression in Caco‐2 cells challenged with *E. coli*. Interestingly, *E. coli* exposure resulted in a significant decrease in *trans*‐epithelial electrical resistance values in Caco‐2 cells, reflecting impaired barrier integrity, which was reverted by *S. officinalis* L. wastewater, and this effect was associated with claudin‐1 and occludin restoration, essential for maintaining intestinal barrier function. Present data confirms the protective effect of *S. officinalis* L. hydrodistillation wastewater in *E. coli*‐induced inflammation, suggesting its potential application in the prevention and/or treatment of intestinal inflammation.

## Graphical abstract

Hydrodistillation of *Salvia officinalis* L. generates wastewater enriched in polyphenolic compounds with biological activity. This study evaluates its anti‐inflammatory effects in vitro using LPS‐stimulated Raw 264.7 macrophages and *E. coli*‐challenged Caco‐2 cells. The phytocomplex reduces NF‐κB activation and pro‐inflammatory cytokines, downregulates COX‐2, and restores intestinal barrier integrity by modulating tight junction proteins, suggesting potential application in preventing intestinal inflammation.

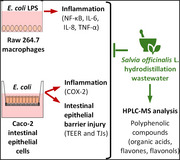

**Abbreviations**
DMEM: Dulbecco's Modified Eagle's Medium
DPBS: Dulbecco's phosphate‐buffered saline
LPS: lipopolysaccharide
TEER: *trans*‐epithelial electrical resistance
TJs: tight junctions

## 1 Introduction

The genus *Salvia* belongs to the *Lamiaceae* family and consists of about 900 species cultivated worldwide and widely employed in traditional medicine and for culinary purposes. Among them, *Salvia officinalis* L. is a perennial, evergreen subshrub highly appreciated for its rich essential oil and its plethora of phytochemicals extensively used in traditional medicine [1]. Based on existing literature, *S. officinalis* exhibits a remarkable chemical diversity in its secondary metabolites, making it one of the most extensively investigated species in the search for new bioactive metabolites [2]. In addition to its widespread use as a culinary herb, consumed both fresh and dried, *S. officinalis* L. can be considered one of the most important aromatic plants intended for the production of essential oil, a phytocomplex of volatile compounds belonging to different chemical families with considerable commercial relevance in perfumery, food additives industry, aromatherapy, and cosmetic fields. The growing importance of essential oils in the last decades is due mainly to their antimicrobial and antiviral activities [3].

Essential oils from medicinal plants are obtained through physical processes such as hydrodistillation and steam distillation. In these processes, the starting material undergoes thermal treatment with water or water vapor. Once the volatile fraction is separated as essential oil, an exhausted residue remains, consisting of water enriched with water‐soluble compounds. Its quantity ranges from approximately 5% of the starting amount in large‐scale industrial plants, to 70%–80% for smaller plants employing closed boilers with water recirculation systems. In all these cases, this solution can be assimilated to the product of a hot aqueous extraction of a vegetal matrix, and therefore rich in all the water‐soluble and thermostable metabolites present in the starting matrix. Distilled water waste is rich in biologically active substances, similar to those obtainable through conventional hot water extraction. The chemical composition of distillation wastewater of several medicinal aromatic plants has been already studied, revealing phytocomplexes rich in biologically active substances, especially of a polyphenolic nature [4, 5, 6], which could be valorized and reused (in compliance with “European green deal” adopted in 2020 by European Commission) in different fields, such as pharmaceutical, cosmetic, nutraceutical and food industries.

Inflammation is a physiological response elicited by the organism against harmful stimuli, including foreign invaders, such as bacteria and viruses, and/or their products [7]. Enteric pathogens, such as *Escherichia coli*, are known to activate an inflammatory process in macrophages, key cells of the first‐line innate immune defense system, and the major cells involved in inflammation, as well as on intestinal epithelium, whose integrity and functionality are essential for maintaining the intestinal barrier [8, 9]. Interestingly, macrophages play several critical roles in intestinal health, significantly contributing to the preservation of gut barrier function [10].

Plant polyphenols have shown potential in the prevention and treatment of inflammatory conditions affecting the gastrointestinal tract [6]. In particular, our previous paper [6] demonstrated that hydrodistillation of wastewaters from aromatic plants of the *Lamiaceae* family exerts protective effects against intestinal inflammation. In fact, when Caco‐2 intestinal epithelial cells were exposed to tumor necrosis factor‐alpha (TNF‐α) as an inflammatory stimulus, treatment with *S. officinalis* hydrodistillation wastewater partially prevented the activation of the nuclear factor kappa B (NF‐κB) pathway and the overexpression of the cyclooxygenase‐2 (COX‐2) protein.

*E. coli* is a common zoonotic foodborne pathogen; it is part of the intestinal commensal flora of humans but can cause gastrointestinal illnesses in humans [11]. In recent years, there has been a significant increase in the incidence of *E. coli* infections, driven by the emergence of drug resistance and the impossibility of a total avoidance of environmental contamination, which represents a major public health concern. *E. coli* infection can activate the innate immune system, triggering the inflammatory cascade and disrupting intestinal barrier function [12, 13].

On this basis, further study has been carried out to better elucidate the cellular and molecular mechanisms potentially involved in the protective effects of *S. officinalis* hydrodistillation wastewater against *E. coli*‐induced inflammation. In these experiments, murine macrophage Raw 264.7 cells and human intestinal epithelial Caco‐2 cells were employed to assess whether pretreatment with *S. officinalis* hydrodistillation wastewater could mitigate the inflammatory response triggered by exposure to *E. coli* lipopolysaccharide (LPS) or *E. coli*, respectively.

## 2 Results

### 2.1 High‐Performance Liquid Chromatography Polyphenolic Profiles of *S. officinalis* L. Hydrodistillation Wastewater

**FIGURE 1:**
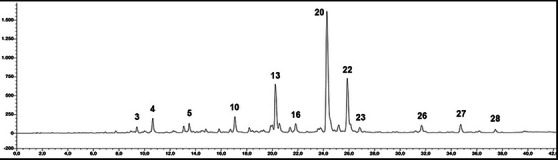
High‐performance liquid chromatography‐ultraviolet/diode array detection (HPLC‐UV/DAD) chromatogram extracted at 330 nm of *Salvia officinalis* L. wastewater. Peaks are numbered as reported in Table 1.

Phytochemical evaluation of the *S. officinalis* L. hydrodistillation wastewater sample was carried out through the high‐performance liquid chromatography (HPLC) identification of the main polyphenolic compounds. Chromatogram at λ = 330 nm is reported as Figure 1, and tentatively identified compounds are listed in Table 1. Our analysis confirms that the distillation wastewater of *S. officinalis* contains a high quantity of polyphenolic compounds per gram of freeze‐dried sample, reaching in our case 48.58% of the total. Most of the identified compounds can be related to the chemical families of organic acids (especially hydroxycinnamic acids), flavones, and, to a lesser extent, flavonols.

**TABLE 1:** Mean values (mg g^−1^ lyophilized powder; analyses were carried out in triplicate) of the major detected polyphenols in the wastewater from *Salvia officinalis* L.

#	Rt	Tentative identification	Identification	mg/g powder (± SD)
1	3.458	Dihydroxyphenylacetic acid	UV, MS	11.04 (± 0.01)
2	5.988	Syringic acid	UV, MS	0.42 (± 0.00)
3	9.124	Hydroxycinnamic acid derivative 1	UV	2.87 (± 0.00)
4	10.168	Hydroxycinnamic acid derivative 2	UV	11.04 (± 0.00)
5	13.607	Hydroxycinnamic acid derivative 3	UV	5.25 (± 0.00)
6	14.019	Apigenin derivative	UV	2.94 (± 0.00)
7	15.067	Apigenin‐6,8‐di‐C‐glycopyranoside	UV, MS	3.57 (± 0.00)
8	15.976	Hydroxycinnamic acid derivative 4	UV	2.05 (± 0.00)
9	16.625	Hydroxycinnamic acid derivative 5	UV	1.89 (± 0.00)
10	17.115	Luteolin derivative 1	UV	18.89 (± 0.01)
11	18.787	Quercetin‐*O*‐glucuronide	UV, MS	4.53 (± 0.00)
12	21.763	Luteolin derivative 2	UV	14.04 (± 0.01)
13	22.046	Luteolin‐O‐glucoside	UV, MS, STD	67.46 (± 0.02)
14	22.176	Luteolin‐*O*‐rutinoside	UV, MS, STD	15.4 (± 0.01)
15	22.426	Luteolin‐X‐O‐glucuronide	UV, MS	5.59 (± 0.01)
16	22.937	Luteolin‐Y‐O‐glucuronide	UV, MS	11.86 (± 0.01)
17	23.348	6‐Methoxyluteolin 7‐glucoside (Nepitrin)	UV, MS	0.95 (± 0.03)
18	23.547	Luteolin derivative 3	UV	3.46 (± 0.01)
19	23.768	Salvianolic acid B	UV, MS	0.6 (± 0.01)
20	24.267	Rosmarinic acid	UV, MS, STD	126.96 (± 0.03)
21	25.008	Apigenin‐7‐glucuronide	UV, MS, STD	8.69 (± 0.01)
22	25.899	Salvianolic acid K	UV, MS	136.03 (± 0.02)
23	26.841	Luteolin derivative 4	UV	5.91 (± 0.00)
24	27.206	Cirsimarin	UV	0.72 (± 0.00)
25	29.761	Unknown	UV	n.a.
26	31.684	Lithospermic acid isomer 1	UV, MS	8.91 (± 0.00)
27	34.74	Salvianolic acid A	UV, MS	10.36 (± 0.01)
28	37.452	Lithospermic acid isomer 1	UV, MS	3.68 (± 0.01)
29	39.726	Cirsimaritin	UV, MS	0.64 (± 0.04)
		**Total phenolics**		485.75
		**Organic acid derivatives**		161.52
		**Salvianolic acids**		159.58
		**Flavones**		160.12
		**Flavonols**		4.53

Rt: retention time (min).

Hydroxycinnamic acids represent 16.5% of the total, with rosmarinic acid as the main compound (peak 20), which reaches a concentration of 127 mg/g. Among organic acids, a prominent chemical subfamily present in our sample is the salvianolic acids, a group of water‐soluble polyphenolic acids consisting of several combinations of caffeic acid and salvianic acid, and produced by many species within the genus *Salvia*. In our sample, the main salvianolic acid is salvianolic acid K, present in the quantity equal to 136 mg/g, followed by a non‐identified salvianolic acid derivative (peak 27, 10.4 mg/g), salvianolic acid A (peak 26, 8.9 mg/g), and salvianolic acid B (peak 28, 3.7 mg/g). Biologically active chemical family of flavones reaches in our sample the percentage of 16% of the total. Ultraviolet (UV) and mass spectrometry (MS) spectra, together with the injection of the relevant analytical standards, allow us to identify peak 13 as luteolin‐O‐glucoside (67.5 mg/g) and peak 14 as luteolin‐O‐rutinoside (15.4 mg/g). Combining UV and LC‐MS data, two luteolin glucuronides have been identified (peaks 15 and 16), while concerning peaks 10, 12, 18, and 23, only addressing their identity as derivatives of luteolin was possible. Relevant were also the quantities of apigenin‐O‐glucuronide (peak 21, 8.7 mg/g) and apigenin‐6,8‐di‐C‐glycopyranoside (Vicenin II, peak 7, 3.6 mg/g). Cirsimarin and its aglycone cirsimaritin (peaks 24 and 29) were detected in trace amounts. The unique flavonol identified in our sample was quercetin‐O‐glucuronide (peak 11, 4.5 mg/g).

### 2.2 Anti‐Inflammatory Effect of *S. officinalis* L. Hydrodistillation Wastewater on Raw 264.7 Macrophages

Firstly, we evaluated the anti‐inflammatory potential of *S. officinalis* L. hydrodistillation wastewater in a cellular model relevant to immune responses. Polyphenols, indeed, are recognized to exert multiple anti‐inflammatory effects by regulating cellular activities and enzymes involved in the inflammatory response, and modulating the production and release of pro‐inflammatory mediators [14]. Macrophages play a crucial role in orchestrating inflammation by recognizing and responding to pathogenic stimuli [15]. Upon exposure to bacterial components such as LPS, macrophages initiate a cascade of pro‐inflammatory signaling events contributing to the amplification of the inflammatory response; if left uncontrolled, this process can result in tissue damage and chronic inflammatory conditions [16].

**FIGURE 2:**
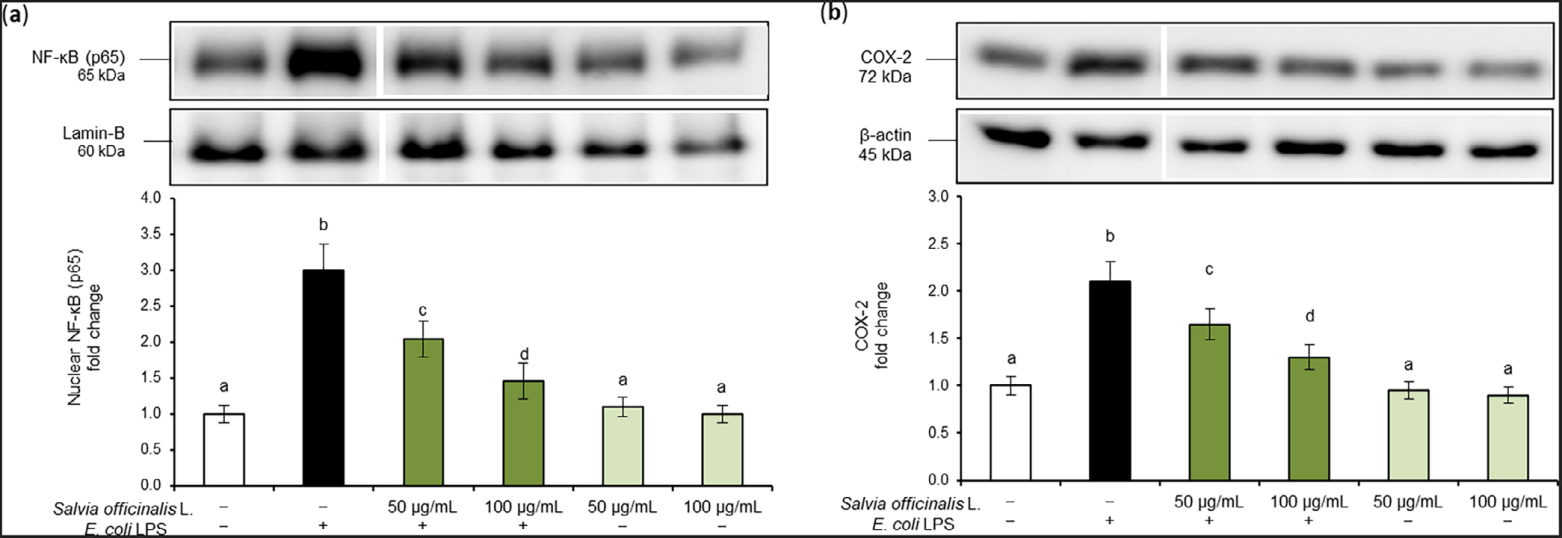
Activation of the nuclear factor kappa B (NF‐κB) pathway. Raw 264.7 cells were pre‐treated for 24 h with *Salvia officinalis* L. hydrodistillation wastewater (50 and 100 µg/mL) and then exposed to *E. coli* lipopolysaccharide (LPS) (100 ng/mL) for 2 h by adding it to the culture medium. Experimental groups included vehicle‐treated cells (used as controls), *E. coli* LPS alone, *E. coli* LPS combined with wastewater pre‐treatment, and wastewater treatment alone. (a) Nuclear p65 (NF‐κB) and (b) cyclooxygenase‐2 (COX‐2) protein levels were analyzed by Western blot. The densitometry results are reported as a fold change against the control. Intensity values were normalized to the corresponding Lamin‐B and β‐actin values. Bands are cropped from original Western blot images for illustrative purposes; the uncropped images are available in the Supplementary Material. Data are presented as mean ± SD from three independent experiments. Statistical analysis was performed using one‐way analysis of variance (ANOVA) followed by Tukey's (Honest Significant Difference [HSD]) post hoc test. Groups sharing the same letter are not significantly different from each other (*p >* 0.05).

In this regard, to further evaluate the anti‐inflammatory activity of *S. officinalis L*. hydrodistillation wastewater, we used an in vitro model of murine macrophage Raw 264.7 cells exposed to *E. coli* LPS and evaluated the effects on the activation of the NF‐κB inflammatory cascade. NF‐κB is a key transcription factor of activated macrophages, also known as M1 macrophages, crucial for the induction of several pro‐inflammatory cytokines [17]. In physiological conditions, NF‐κB subunit p65 is located in the cytoplasm, bound to an inhibitory protein. Once activated by pro‐inflammatory stimuli, p65 becomes free to translocate into the nucleus, where it activates the transcription of different inflammatory genes [18]. In this experimental model, cells exposed to *E. coli* LPS (100 ng/mL) showed increased nuclear translocation of p65 subunit (Figure 2a) and release of COX‐2 cytoplasmic pro‐inflammatory enzyme (Figure 2b). On the contrary, these markers were inhibited by pre‐treatment with *S. officinalis* L. hydrodistillation wastewater in a dose‐dependent manner. Treatments did not alter total p65 (NF‐κB) (see Figure S2).

**FIGURE 3:**
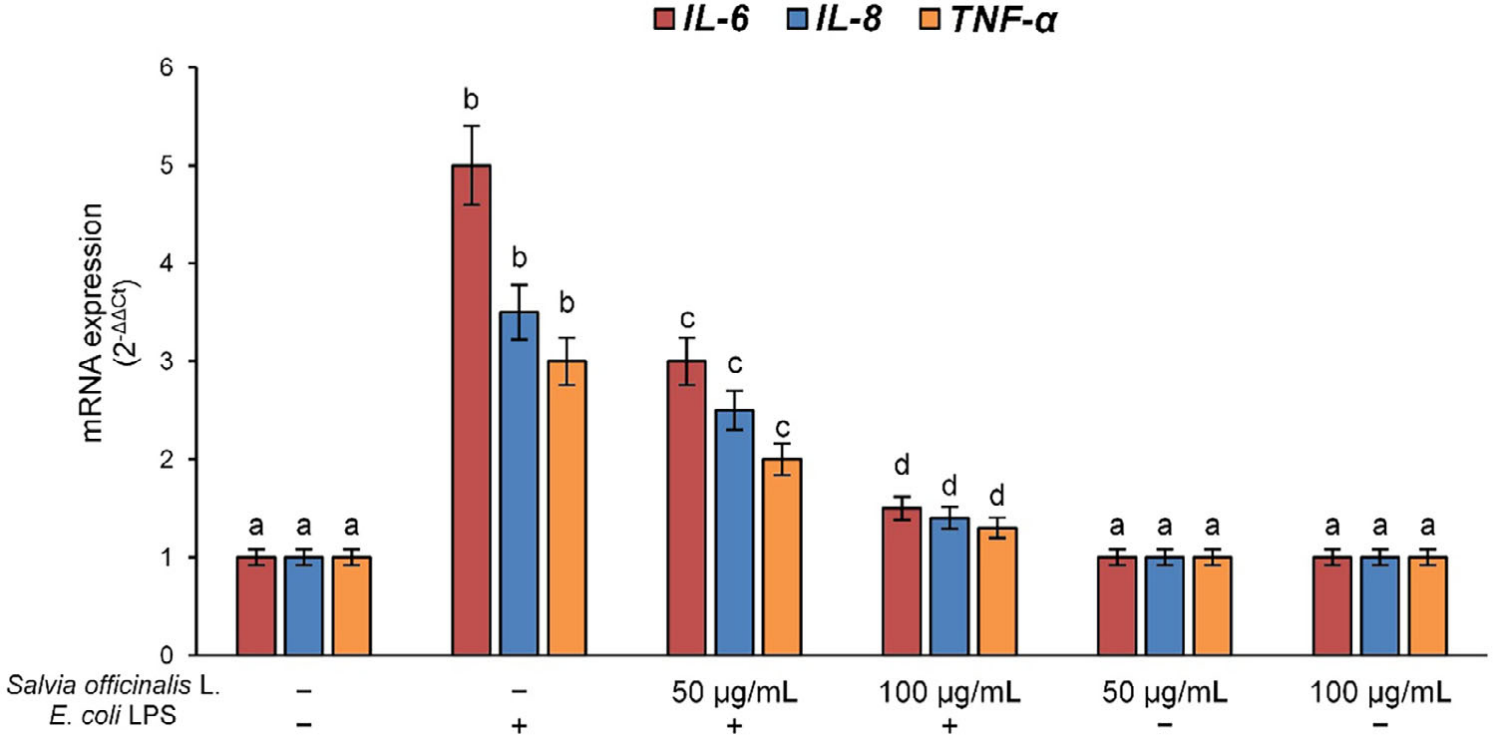
Effects on pro‐inflammatory cytokines gene expression. Raw 264.7 cells were pre‐treated for 24 h with *Salvia officinalis* L. hydrodistillation wastewater (50 and 100 µg/mL) and then exposed to *E. coli* lipopolysaccharide (LPS) (100 ng/mL) for 2 h by adding it to the culture medium. Experimental groups included: vehicle‐treated cells (used as controls), *E. coli* LPS alone, *E. coli* LPS combined with wastewater pre‐treatment, and wastewater treatment alone. Interleukin (*IL*)*‐6*, *IL‐8*, and tumor necrosis factor (*TNF*)*‐α* mRNA values are expressed as 2^‐ΔΔCt^ and normalized to control. 18S rRNA was used as a housekeeping gene. Data are presented as mean ± SD from three independent experiments. Statistical analysis was performed using one‐way analysis of variance (ANOVA) followed by Tukey's (Honest Significant Difference [HSD]) post hoc test. Groups sharing the same letter are not significantly different from each other (*p >* 0.05).

Therefore, to confirm the NF‐κB transcriptional activity, we evaluated the expression of interleukin (*IL*)*‐6, IL‐8*, and *TNF‐α* genes, the major cytokines involved in the activation of immune cells and diffusion of inflammation. Results demonstrated *E. coli* LPS's ability to induce an overexpression of these pro‐inflammatory genes in Raw 264.7 cells. Also in this case, pre‐treatment with *S. officinalis* L. hydrodistillation wastewater was able to dose‐dependently prevent the activation of the inflammatory cascade (Figure 3).

Overall, all results confirm the anti‐inflammatory activity of *S. officinalis* L. hydrodistillation wastewater in *E. coli* LPS‐induced inflammation, demonstrating the beneficial potential of these natural compounds in human health.

### 2.3 *S. officinalis* L. Hydrodistillation Wastewater Inhibits COX‐2 Expression Induced by *E. coli* in Caco‐2 Cells

Given the protective effects observed in the macrophages exposed to *E. coli* LPS, we next investigated the effect of *S. officinalis* L. hydrodistillation wastewater in an intestinal epithelial model. Inflammatory responses triggered by enteric pathogens such as *E. coli* often involve upregulation of COX‐2, an inducible enzyme responsible for the production of pro‐inflammatory prostaglandins [18]. Increased COX‐2 expression is typically associated with intestinal epithelial damage and alterations in permeability, primarily due to changes in tight junctions (TJs) assembly [19, 20, 21, 22].

**FIGURE 4:**
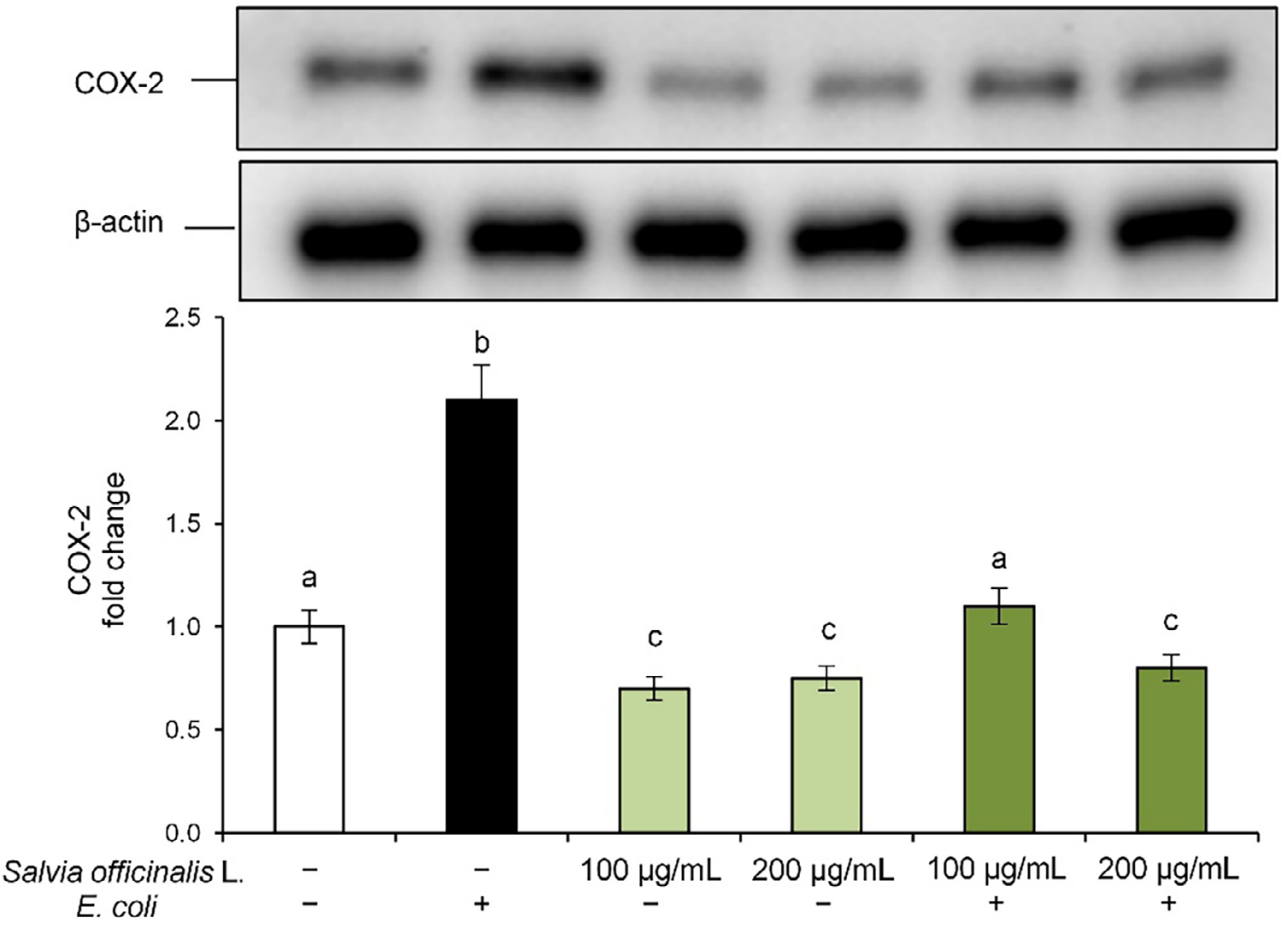
Modulation of cyclooxygenase‐2 (COX‐2) expression. Caco‐2 cells were pre‐treated with *Salvia officinalis* L. hydrodistillation wastewater (100 and 200 µg/mL) for 24 h and subsequently exposed to *E. coli* for 2 h. Experimental groups included: vehicle‐treated cells (used as controls), *E. coli* alone, wastewater treatment alone, and *E. coli* combined with wastewater pre‐treatment. COX‐2 protein level was analyzed by Western blot. The densitometry results are reported as a fold change against the control. COX‐2 intensity value was normalized to the corresponding β‐actin value. Data are presented as mean ± SD from three independent experiments. Statistical analysis was performed using one‐way analysis of variance (ANOVA) followed by Tukey's (Honest Significant Difference [HSD]) post hoc test. Groups sharing the same letter are not significantly different from each other (*p >* 0.05).

In our experimental model, *E. coli* exposure led to a significant increase in COX‐2 protein expression compared to control cells, indicating the onset of an inflammatory state in Caco‐2 cells (Figure 4). However, pre‐treatment with *S. officinalis* L. hydrodistillation wastewater effectively downregulated COX‐2 expression in a dose‐dependent manner, reaching even lower levels than the control at the highest tested concentration, thus confirming its anti‐inflammatory potential. Notably, treatment with *S. officinalis* L. hydrodistillation wastewater alone also reduced baseline COX‐2 levels, further supporting its role in modulating inflammation.

### 2.4 Protective Effect of *S. officinalis* L. Hydrodistillation Wastewater on Intestinal Barrier Integrity Altered by *E. coli*

Intestinal epithelium plays an essential role in maintaining gut homeostasis by acting as a dynamic and selectively permeable barrier that regulates the passage of essential nutrients, while simultaneously preventing potentially harmful agents from entering the systemic circulation [23]. A well‐established approach for assessing epithelial integrity in vitro involves measurement of *trans*‐epithelial electrical resistance (TEER). A reduction in TEER values, in fact, reflects impairment of barrier function, often associated with increased paracellular permeability resulting from pathogenic infections [24].

**FIGURE 5:**
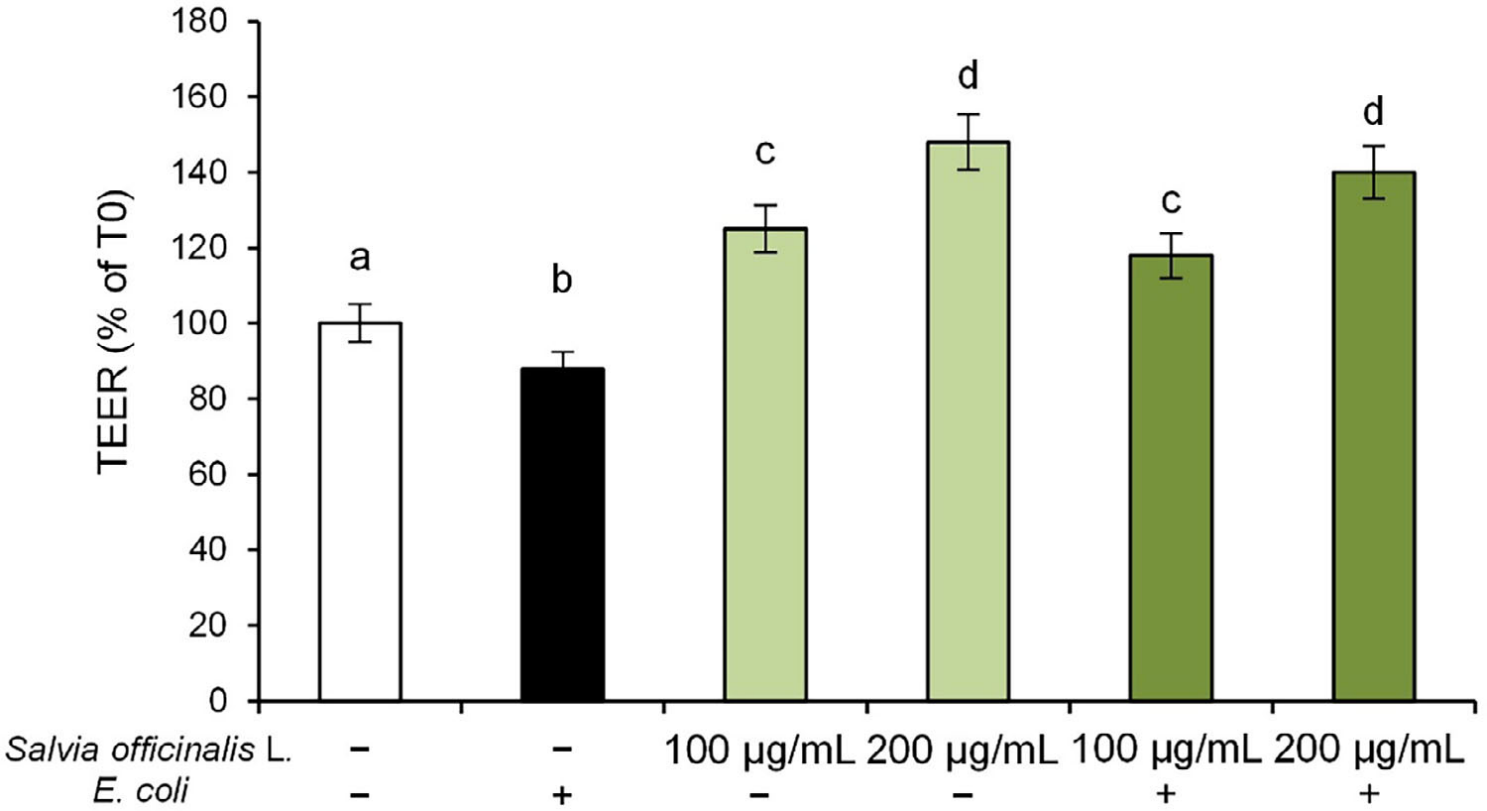
*trans*‐Epithelial electrical resistance (TEER) of Caco‐2 cells. Caco‐2 cells were pre‐treated with *Salvia officinalis* L. hydrodistillation wastewater (100 and 200 µg/mL) for 24 h and subsequently exposed to *E. coli* for 2 h. Experimental groups included: vehicle‐treated cells (used as controls), *E. coli* alone, wastewater treatment alone, and *E. coli* combined with wastewater pre‐treatment. The TEER values were measured before bacterial exposure (time zero, T0) and at the end of the treatment (T2). Results are reported as fold change of values at T2 relative to values at T0 and presented as mean ± SD from three independent experiments. Statistical analysis was performed using one‐way analysis of variance (ANOVA) followed by Tukey's (Honest Significant Difference [HSD]) post hoc test. Groups sharing the same letter are not significantly different from each other (*p >* 0.05).

Therefore, in order to investigate the protective effects of *S. officinalis* L. hydrodistillation wastewater on Caco‐2 monolayers exposed to *E. coli*, TEER was measured after 2 h of pathogenic bacterium exposure. The results showed that *E. coli* exposure resulted in a significant decrease in TEER values compared to control cells, reflecting impaired barrier integrity (Figure 5). However, pre‐treatment with *S. officinalis* L. hydrodistillation wastewater counteracted this effect, leading to a significant dose‐dependent increase in TEER values compared to CTR cells, suggesting a potential role in preserving barrier integrity.

Notably, treatment with *S. officinalis* L. hydrodistillation wastewater alone also improved baseline TEER values in a dose‐dependent manner, suggesting a potential role in enhancing barrier integrity.

This finding suggests that the bioactive compounds present in the hydrodistillation wastewater may contribute to the maintenance of epithelial barrier function, potentially through modulation of inflammatory signaling pathways at the cellular level.

### 2.5 *S. officinalis* L. Hydrodistillation Wastewater Restores TJ Proteins Expression in *E. coli*‐Challenged Caco‐2 Cells

**FIGURE 6:**
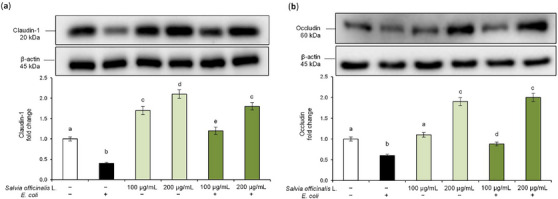
Modulation of tight junction protein expression. Caco‐2 cells were pre‐treated with *Salvia officinalis* L. hydrodistillation wastewater (100 and 200 µg/mL) for 24 h and subsequently exposed to *E. coli* for 2 h. Experimental groups included: vehicle‐treated cells (used as controls), *E. coli* alone, wastewater treatment alone, and *E. coli* combined with wastewater pre‐treatment. Claudin‐1 (a) and occludin (b) protein levels were analyzed by Western blot. The densitometry results are reported as a fold change against the control. Claudin‐1 and occludin intensity values were normalized to the corresponding β‐actin value. Data are presented as mean ± SD from three independent experiments. Statistical analysis was performed using one‐way analysis of variance (ANOVA) followed by Tukey's (Honest Significant Difference [HSD]) post hoc test. Groups sharing the same letter are not significantly different from each other (*p >* 0.05).

TJs proteins are key components of the epithelial barrier, controlling paracellular transport and maintaining tissue integrity [25]. Based on the TEER results, we further evaluated the effect of the hydrodistillation wastewater on the modulation of TJs proteins altered by *E. coli*. Specifically, we analyzed claudin‐1 and occludin, which are essential for preserving intestinal barrier function and for stabilizing cell‐cell interactions [26]. Disruption of these proteins, in fact, is a hallmark of inflammatory processes and bacterial infections [27]. Western blot analysis revealed that *E. coli* infection significantly reduced claudin‐1 and occludin protein levels in Caco‐2 cells compared to control cells, confirming the pathogen‐induced disruption of TJs (Figure 6). Interestingly, pre‐treatment with *S. officinalis* L. hydrodistillation wastewater restored and even enhanced claudin‐1 and occludin expression in a dose‐dependent manner. Moreover, treatment with *S. officinalis* L. hydrodistillation wastewater alone also led to an increase in baseline claudin‐1 and occludin levels. These findings suggest that *S. officinalis* L. hydrodistillation wastewater exerts a protective effect on the intestinal epithelium against *E. coli*‐induced damage by enhancing the expression of TJs proteins and improving barrier integrity. Overall, these findings support the hypothesis that hydrodistillation wastewater preserves intestinal barrier integrity against *E. coli*‐induced intestinal epithelial injury, most likely through anti‐inflammatory mechanisms.

## 3 Discussion

Plants belonging to the genus *Salvia* are mainly used in ethnopharmacology in the form of decoctions prepared from aerial parts or roots to treat microbial infections, cancer, malaria, inflammation, memory impairment, and even for domestic disinfection following illness [28]. Such applications are probably justified by the high content of biologically active substances, in particular polyphenols, present in the aqueous extracts. Consequently, in recent years, increasing scientific attention has been directed toward the water‐soluble components of these folk preparations, given their potential pharmacological relevance [29]. The residual waters obtained from the distillation of aromatic plants in general can, to some extent, be considered analogous to a decoction, as both involve hot water extraction. It is therefore reasonable to assume that even in this waste there are biologically active substances, water‐soluble and thermostable, which, once recovered, may exhibit diverse biological activities. This assumption is supported by several investigations conducted on species such as lavender [5, 30], basil [31], rosemary, thyme, and sage [6, 31]. Collectively, these studies confirmed the presence of phenolic acids (mainly derivatives of caffeic acid and rosmarinic acid), along with phenolic glycosides, flavones, and flavonols in the residual distillation waters, present in variable but consistently significant amounts.

In our sample, chemical profiling of the phenolic fraction reveals a phytocomplex characterized by a high content of polyphenols (48.58%), almost equally distributed across three subclasses: organic acid derivatives, salvianolic acids, and flavones. Among the others, salvianolic acids account for up to 15.96% of the total phenolic composition. These compounds, previously reported in sage infusions [31, 32, 33], are water‐soluble phenolic compounds recognized for their strong antioxidant activity [34], deriving from several structural combinations of caffeic acid and salvianic acid. In our sample, salvianolic acid K, salvianolic acid A, two isomers of lithospermic acid, and salvianolic acid B have been detected. Salvianolic acids (especially salvianolic acid A and B) are the main compounds of *Salvia milthiorrizha* extracts, which have been traditionally employed in Chinese folk medicine to treat cardiovascular‐related diseases in China and other Asian countries for centuries. Several studies have demonstrated that the cardiovascular protective effects of salvianolic acids are mediated by multiple mechanisms, including reduction of leukocyte‐endothelial adherence, inhibition of inflammatory processes, and downregulation of metalloproteinases expression in aortic smooth muscle cells, together with the competitive binding to target proteins, disrupting critical protein‐protein interactions [35]. Recent studies have demonstrated that these compounds also exert beneficial effects on the alleviation of fibrosis disease and the treatment of cancer through their ability to modulate signal transduction pathways within fibroblasts and cancer cells. It is demonstrated that salvianolic acids promote apoptosis in cancer cells and inhibit cancer‐associated epithelial‐mesenchymal transition processes [36]. However, Kim et al. [37] reported that a water‐soluble extract from *Salvia miltiorrhiza* roots, but not its constituent salvianolic acid B, abrogates LPS‐induced NF‐κB signaling in intestinal epithelial cell culture in vitro. Furthermore, the polycyclic phenolic carboxylic acid lithospermic acid, as well as the dimethoxyflavone cirsimaritin, has been shown to protect mice against dextran sulfate sodium‐induced colitis through NF‐κB inhibition and nuclear factor erythroid 2‐related factor 2 (Nrf2) signaling pathway activation [38, 39]. In addition, lithospermic acid alleviates oxidative stress and inflammation in LPS‐stimulated normal human colon mucosal epithelial NCM460 cells [39].

The main phenolic compound in our sample is rosmarinic acid, an ester between caffeic acid and 3,4‐dihydroxyphenyl lactic acid. It is a phenolic compound widely distributed throughout the plant kingdom, especially in species of the Lamiaceae family, and is well known for its considerable potential in therapeutic and food applications owing to its biological properties, such as antioxidant, anti‐inflammatory, neuroprotective, chemopreventive, cardioprotective, and antidiabetic effects, among others [40]. Rosmarinic acid also plays a significant role in the management of inflammatory diseases through multiple mechanisms, as reported by Luo et al. [41], and exhibits promising anticancer activity together with its derivatives, salvianolic acids [42]. Moreover, recent evidence in murine models indicates that rosmarinic acid alleviates *E. coli*‐induced inflammatory damage to the intestinal barrier by inhibiting the NF‐κB signalling pathway and preserving gut microbiota homeostasis [43].

The other main chemical family in our sage sample consists of flavones, mainly represented by luteolin derivatives. Luteolin is a bioactive polyphenolic flavone with various health‐promoting properties, including anti‐hypertensive, anti‐diabetic, anti‐asthmatic, anti‐carcinogenic, as well as anti‐viral properties [44]. Several experimental results demonstrated a notable anti‐inflammatory effect, both in vitro and in vivo, through the interaction of luteolin and luteolin‐O‐glucoside [45] with JAK/STAT3, NF‐κB, and other cell signaling pathways. Another less studied derivative, luteolin‐O‐glucuronide, also showed anti‐inflammatory activity by inhibiting nitric oxide and pro‐inflammatory cytokines production [46], together with a promising antiviral action against severe acute respiratory syndrome coronavirus 2, as reported in a recent in silico study [47].

In the second part of our research, the experiments were designed to explore the potential protective effects of *S. officinalis* L. hydrodistillation wastewater against intestinal inflammation and to clarify the possible mechanisms responsible for this activity. To reach this goal, two in vitro models were employed: *E. coli* LPS‐stimulated RAW 264.7 macrophage cells and *E. coli*‐exposed Caco‐2 cell monolayers. *S. officinalis* L. hydrodistillation wastewater concentration ranges used in this study (50 and 100 µg/mL in RAW 264.7 cells; and 100 and 200 µg/mL Caco‐2 cells) are consistent with those commonly employed in cell‐based assays and may reflect achievable local concentrations in the gastrointestinal tract or inflamed tissues following dietary or nutraceutical intake [48, 49, 50, 51].

Macrophages are the main pro‐inflammatory cells and have a central function in protecting the organism from external agents. When activated, macrophages release large amounts of pro‐inflammatory mediators such as cytokines. The transcription factor NF‐κB plays an important role in the transcription of genes involved in inflammatory responses, including *TNF‐α, IL‐1β*, and *IL‐6* genes. Our findings showed that exposure of RAW 264.7 macrophages to *E. coli* LPS increased NF‐κB nuclear translocation and COX‐2 pro‐inflammatory enzyme levels, and triggered a marked overexpression of *IL‐6*, *IL‐8*, and *TNF‐α* genes. Pre‐treatment with *S. officinalis* L. hydrodistillation wastewater prevented LPS‐induced activation of the inflammatory cascade in a dose‐dependent manner, normalizing both NF‐κB nuclear translocation and *IL‐6, IL‐8*, and *TNF‐α* overexpression.

The gut epithelium represents a physical barrier that mediates selective permeability to exogenous agents. In our study, a Caco‐2 cell monolayer was used as an intestinal epithelial barrier model, and *E. coli* to simulate in vitro a condition of intestinal injury caused by enteropathogen exposure. In this model, the extract, along with *E. coli*, was applied to the apical side of the Caco‐2 monolayer to simulate oral administration of the *S. officinalis* L. hydrodistillation wastewater and the presence of the bacterial strain in the intestinal lumen.

Initially, to clarify the effects on the inflammatory response, we evaluated the expression of the inducible enzyme COX‐2, responsible for the production of pro‐inflammatory prostaglandins [22]. In this experimental model, E. coli exposure induced a marked increase in COX‐2 protein levels in Caco‐2 cells, whereas pretreatment with the *S. officinalis* L. hydrodistillation wastewater suppressed, in a dose‐dependent manner, COX‐2 expression induced by *E. coli*, highlighting the anti‐inflammatory effect of this product.

Then, we focused on the protective effects of *S. officinalis* L. hydrodistillation wastewater on *E. coli*‐induced disruption of intestinal epithelial barrier function. The decrease in TEER values confirmed that *E. coli* exposure compromises the epithelial barrier. Furthermore, our findings confirmed that *E. coli* exposure can affect intestinal epithelium integrity, through reduction of claudin‐1 and occludin, the main apical junction proteins that connect epithelial cells and so regulate the transport of molecules from the intestinal lumen to the bloodstream. The protective effect of *S. officinalis* L. hydrodistillation wastewater against *E. coli*‐induced damage to monolayer integrity was clearly evidenced by the significant and dose‐dependent increase in TEER values as well as in TJs protein levels. Moreover, the *S. officinalis* L. hydrodistillation wastewater increased TEER values and TJs expression even in cells not exposed to *E. coli*, suggesting its ability to exert a beneficial effect on intestinal epithelial barrier function. Since the increased expression of COX‐2 may be related to changes in TJs assembly and consequently to impairment of intestinal epithelial barrier function [52], the protective effect of S*. officinalis* L. hydrodistillation wastewater against *E.coli*‐induced damage might be due, at least in part, to the reduction of the inflammatory response triggered by pathogen challenge.

Our findings are consistent with previous studies on hydrodistillation wastewaters from Lamiaceae species, which have demonstrated significant antioxidant and anti‐inflammatory properties associated with their rich phenolic profiles. In particular, in our earlier work [6], we reported that wastewaters from Lamiaceae exhibited comparable biological activities, supporting the hypothesis that these by‐products represent a valuable source of bioactive compounds. Compared to leaf extracts of *S. officinalis* L., which are traditionally studied for their pharmacological properties [53, 54], the wastewater investigated here shows similar protective effects, highlighting its potential for sustainable recovery and application in nutraceutical and pharmaceutical formulations.

Altogether, the present data confirms the protective effect of *S. officinalis* L. hydrodistillation wastewater against bacterial‐induced intestinal inflammation, suggesting a potential application of this product in the prevention and/or treatment of intestinal inflammation. It is evident that, due to the potential efficacy in preventing *E. coli*‐related intestinal damage in humans, the development of this phytocomplex, rich in bioactive compounds and obtained as a byproduct of plant extraction, could have an important impact on human health with significant social and economic implications. At the current state of our studies, however, it is not possible to hypothesize which of the components of *S. officinalis* L. wastewater might be responsible for the biological activity demonstrated or to what extent; however, it is conceivable that they exert a synergistic action. More insights into this aspect could be useful to improve the hydrodistillation process, enhancing its efficiency and cost‐effectiveness. Nevertheless, this work was conceived as an initial mechanistic investigation using in vitro models, which are widely recognized for preliminary screening of bioactive compounds. While these models provide valuable insights into cellular and molecular pathways, they cannot fully replicate the complexity of an intact organism. Therefore, the absence of in vivo validation represents a limitation that may affect the translational relevance of our findings. Future studies employing animal models, such as zebrafish or murine models of colitis, are warranted to confirm and extend these observations.

## 4 Materials and Methods

### 4.1 Chemicals

HPLC grade solvents were from Honeywell (Milan, Italy), reference standards were purchased from Sigma‐Aldrich Products (Merck KGaA, Darmstadt, Germany. LPS from *Escherichia coli* O127:B8 was obtained from Sigma‐Aldrich (Milan, Italy). All other reagents, unless otherwise specified, were acquired from Sigma‐Aldrich (Milan, Italy).

### 4.2 Plant Material

The studied material was obtained from *S. officinalis* L. plants cultivated in Aragona (Agrigento, Italy) and kindly provided by Rinoldo Davide s.r.l. Samples of the whole plant have been collected in the balsamic period (May‐June) of 2020 and identified by the vendor (voucher specimen 001/2020 kept at the Institute of Biomolecular Chemistry‐ Catania herbarium).

### 4.3 Plant Hydrodistillation and Wastewater Recovery

Fresh plant has been dried at room temperature up to constant weight. Leaves of *S. officinalis* L. have been manually separated from the whole plant, and 100 g of air‐dried material were hydrodistilled with 1 L of distilled water as previously described [6].

### 4.4 Wastewaters HPLC Analysis

The sample for analysis was a solution of 10 mg of lyophilized wastewater in 1 mL of HPLC‐grade water. This solution was filtered on PTFE 0.45 µm filters (PALL Corporation, Port Washington, USA) and put into 2 mL amber glass vials. HPLC analysis for polyphenols identification and quantification was carried out on an Ultimate3000 instrument using a photodiode array as detector (Thermo Scientific, Rome, Italy). For all chromatographic runs, a reverse‐phase column was used (Gemini C18, 250 × 4.6 mm, 5 mm particle size, guard column Gemini C18 4 × 3.0 mm, 5 mm, Phenomenex, Rome, Italy). The elution program was a gradient of 5%–90% solution B (2.5% formic acid in acetonitrile) in solution A (2.5% formic acid in water) over 50 min, then 7 min at 100% of solution B, with a constant solvent flow rate of 1 mL/min. The quantification was carried out through the construction of calibration curves in HPLC‐UV‐DAD using standards with a similar chromophore to the compound to be quantified, and where the molecular mass was not available, referring to the quantification as equivalent to the reference standard used in the same calibration curve. More in details, quantification was carried out at 330 nm using caffeic acid (*y* = 1E+07*x* + 1.1421, r^2^ = 0.988, limit of detection [LOD] = 1.695 × 10^−7^, limit of quantification [LOQ] = 5.137 × 10^−7^) and rosmarinic acid (*y* = 2E+07*x* – 0.0685, r^2^ = 0.998, LOD = 1.9280 × 10^−6^, LOQ = 5.8425 × 10^−6^) for phenolic acids, luteolin‐7‐O‐glucoside (*y* = 3E+07*x* – 0.0982, r^2^ = 0.999, LOD = 2.640 × 10^−6^, LOQ 7.999 × 10^−6^) as standards for flavones. Calculated LOD and LOQ were expressed in mol, considering S/N 3.3 and 10, respectively. To confirm peak assignment, a series of HPLC/ESI‐MS analyses was carried out. The HPLC apparatus, solvent system, and elution programs used were the same as described above, while ESI mass spectra were acquired as already reported [55].

### 4.5 Cell Cultures and Treatments

#### 4.5.1 Bacterial Strain and Culture Conditions

In this study, the enteropathogenic strain *E. coli* O157:H7 (ATCC 43888) was grown on Tryptic Soy Agar (TSA, Oxoid, Italy) under a 5% CO_2_ atmosphere at 37°C for 24 h.

#### 4.5.2 Raw 264.7 Cells

Murine macrophage Raw 264.7 cells were obtained from American Tissue Culture Collection (ATCC, Manassas, VA, USA) (ATCC Number: TIB‐71; RRID: CVCL_0493) and cultured in Dulbecco's Modified Eagle's Medium (DMEM), supplemented with 10% fetal bovine serum (FBS), 4 mM L‐glutamine, and 100 U/mL penicillin/streptomycin. Cells were maintained at 37°C in a humidified atmosphere with 95% air and 5% CO_2_. For experiments, Raw 264.7 cells were plated into 12‐well plates (Greiner Bio‐One, Italy) for evaluating gene expression, and in 6‐well plates (Greiner Bio‐One, Italy) for protein analysis, using a density of 3 × 10^4^/cm^2^. One day after cell seeding, Raw 264.7 cells were pre‐treated with *S. officinalis* L. hydrodistillation water (50 and 100 µg/mL) for 24 h. Wastewater concentrations were selected based on preliminary cytotoxicity data obtained via Sulforhodamine B (SRB) assay, which showed no significant cytotoxic effects up to 100 µg/mL in Raw 264.7 cells (see Figure S1). Accordingly, 100 µg/mL was chosen as the maximum concentration for subsequent experiments. The sample was always dissolved in DMEM immediately before use. Then, cells were exposed to *E. coli* LPS for 2 h by adding it to the culture medium at a concentration of 100 ng/mL. *E. coli* LPS was dissolved in sterile‐filtered water before use. Raw 264.7 cells treated with the vehicle alone (DMEM) were used as controls. At the end of appropriate treatments, cells have been immediately processed and/or preserved until analysis as expected for each test.

#### 4.5.3 Caco‐2 Cells

Human intestinal Caco‐2 cells, obtained from the American Type Culture Collection (ATCC, Manassas, VA, USA) (ATCC Number: HTB‐37; RRID: CVCL_0025), were cultured in DMEM supplemented with 10% (v/v) heat‐inactivated FBS, 4 mM L‐glutamine, 100 µg/mL streptomycin, 100 U/mL penicillin, and 1% (v/v) non‐essential amino acids. Cells were maintained at 37°C in a humidified incubator with 5% CO_2_ and 95% air. For differentiation, Caco‐2 cells were seeded at a density of 4 × 10⁴ cells/cm^2^ onto the apical side of transwell inserts (0.4 µm pore size; Greiner Bio‐One, Italy) and cultured under the same conditions for 18 days. Complete differentiation was verified by measuring TEER using a Millicell‐ERS Voltohmmeter (Millipore, MA, USA), with only monolayers exhibiting TEER values of ≥600 Ω × cm^2^ included in the experiments. The culture medium was refreshed every 2–3 days.

Before *E. coli* exposure, Caco‐2 monolayers were incubated in antibiotic‐free medium for 16 h to prevent any potential interference with bacterial growth and washed twice with Dulbecco's phosphate‐buffered saline (DPBS, pH 7.4) to remove residual antibiotics and non‐adherent cells [56].

Bacterial cultures were harvested by centrifugation at 4500 × g for 10 min, and the resulting pellet was washed twice with DPBS (pH 7.4) to eliminate any remaining culture medium. The bacterial cells were then resuspended in 1 mL of serum‐ and antibiotic‐free DMEM, supplemented only with 4 mM L‐glutamine and 1% (v/v) non‐essential amino acids, to reach a final concentration of approximately 10⁶–10⁷ CFU/mL before use in the experiments.

To evaluate the *S. officinalis* L. hydrodistillation wastewater's ability to inhibit the *E. coli* effects on Caco‐2 cells, the hydrodistillation wastewater was added to the apical chamber of fully differentiated Caco‐2 monolayers for 24 h. Following this pre‐treatment, Caco‐2 cells were washed with DPBS, and a suspension of *E. coli* was added to the upper chamber, and the incubation was carried out for 2 h under the same conditions. Caco‐2 cells treated with the vehicle alone (serum‐ and antibiotic‐free DMEM) were used as controls. In all experiments, the *S. officinalis* L. hydrodistillation wastewater (100 and 200 µg/mL) was freshly dissolved in medium immediately before use. Wastewater concentrations were selected based on preliminary cytotoxicity data obtained via SRB assay, which showed no significant cytotoxic effects up to 200 µg/mL in Caco‐2 cells (see Figure S1). Accordingly, 200 µg/mL was chosen as the maximum concentration for subsequent experiments.

Following the treatments, barrier integrity was assessed by TEER measurement. Subsequently, the cells have been immediately processed and/or preserved until analysis as expected for each test.

### 4.6 Assessment of in Vitro Barrier Function

The integrity of the intestinal epithelial monolayer was assessed throughout the experimental treatments by measuring TEER using a Millicell‐ERS Voltohmmeter (Millipore, MA, USA). Specifically for TEER analysis, measurements were recorded at the beginning of the *E. coli* challenge (T0) and monitored over the following 2 h (T2). Caco‐2 cells not exposed to bacteria or hydrodistillation wastewater served as controls. TEER values were expressed as fold change relative to the baseline measurements at T0.

### 4.7 Cell Lysates Extraction

At the end of the treatments, cytoplasmatic and nuclear proteins were isolated from RAW 264.7 cells, whereas total protein extraction was carried out for Caco‐2 cells.

In more detail, Raw 264.7 cells were first incubated with a hypotonic buffer (10 mM HEPES, pH 7.9, 10 mM KCl, 1.5 mM MgCl_2_, 5% glycerol) to separate cytoplasmatic proteins, and subsequently incubated with a hypertonic buffer (20 mM HEPES, pH 7.9, 1 mM MgCl_2_, 400 mM NaCl, 1 mM EGTA, 0.1 mM EDTA, 10% glycerol) for nuclear ones. The lysis buffer contained protease inhibitors (1 µg/mL leupeptine, 2 µg/ mL aprotinine, 1 mM benzamidine, and 5 mM NaF) and 1 mM dithiothreitol. Cytoplasmatic and nuclear protein fractions were then stored at ‐80°C until further analysis.

For Caco‐2 cells, total protein extraction was performed by rinsing the cells with PBS and lysing them in a buffer containing 10 mM Tris‐HCl, 150 mM NaCl, 5 mM EDTA Na_2_, and 0.1% (v/v) Triton. The lysates were incubated for 30 min at 4°C to ensure complete protein solubilization and subsequently stored at ‐80°C until further analysis.

Protein concentration in each lysate was determined using the Bradford reagent [57], with bovine serum albumin as a standard.

### 4.8 Western Blot Analysis

For immunoblotting studies, 30 µg of total protein lysates or 10 µg of nuclear ones per sample were denatured and subjected to sodium dodecyl sulfate–polyacrylamide gel electrophoresis as previously described [58]. Afterwards, proteins were transferred to a PVDF membrane (Hybond‐P PVDF, Amersham Bioscience). Then, residual binding sites on membranes were blocked by incubation with 5% (w/v) non‐fat milk powder dissolved in TBST (10 mM Tris, 100 mM NaCl, 0.1% Tween 20) for 1 h at room temperature. Membranes were incubated overnight at 4°C with specific primary antibodies: rabbit anti‐claudin‐1 (1:2000) monoclonal antibody (Cell Signaling Technology); mouse anti‐occludin (1:500) monoclonal antibody (Santa Cruz Biotechnology); mouse anti‐COX‐2 monoclonal antibody (Santa Cruz Biotechnology) (1:200); rabbit anti‐NF‐κB p65 monoclonal antibody (Cell Signaling Technology) (1:1000); rabbit anti‐β‐actin monoclonal antibody (Cell Signaling Technology) (1:6000) and rabbit anti‐Lamin‐B monoclonal antibody (Cell Signaling Technology) (1:500). Later, membranes were exposed to peroxidase‐conjugated secondary anti‐rabbit Ig (Cell Signaling Technology, Danvers, MA, USA) (1:6000) or goat anti‐Mouse IgM Secondary Antibody, HRP conjugate (Cell Signaling Technology) (1:6000) for 2 h at 4°C, and luminescence was visualized with Clarity Max System (Bio‐Rad, Hercules, CA, USA). The protein loading was evaluated by Ponceau S staining and by housekeeping proteins β‐actin and Lamin‐B. Quantitative measurement was performed by Image Lab software (Bio‐Rad, Hercules, CA, USA).

### 4.9 Real‐Time Polymerase Chain Reaction

Total cellular RNA was extracted by E.Z.N.A. Total RNA Kit I (OMEGA Bio‐Tek VWR, Invitrogen), following the manufacturer's instructions, then quantified using Quant‐iTRNA assay kit by QUBIT fluorometer (Invitrogen, Milan, Italy), and finally reverse transcribed with M‐MLV reverse transcriptase. Real‐Time Polymerase Chain Reaction (PCR) was performed on 7300 Real‐Time PCR System (Applied Biosystems, Monza, Italy) using SYBR green chemistry (SYBR green JumpStart Taq Ready Mix, Sigma). The following primers were used: IL‐6 (FW 5′‐GAT GGA TGC TAC CAA ACT GGA T‐3′, RV 5′‐CCA GGT AGC TAT GGT ACT CCA GA‐3′) [59], IL‐8 (FW 5′‐ GCA CTT GGG AAG TTA ACG CA‐3′, RV 5′‐ GCA CAG TGT CCC TAT AGC CC‐3′) [60], TNF‐α (FW 5′‐AAG CCT GTA GCC CAC GTC GTA‐3′, RV 5′‐GGC ACC ACT AGT TGG TTG TCT TTG ‐3’) [60], 18S rRNA (FW 5′‐ GCA CTT GGG AAG TTA ACG CA‐3′, RV 5′‐ GCA CAG TGT CCC TAT AGC CC‐3′) [61]. The fold increase of mRNA expression, compared with the control cells and corrected with 18S rRNA as housekeeping gene, was determined using the 2^−ΔΔCt^ method [62].

### 4.10 Statistical Analysis

All experiments were conducted in triplicate and repeated three times. Data are presented as mean ± standard deviation (SD) from three independent experiments. Statistical comparisons were performed using one‐way analysis of variance, followed by Tukey's Honest Significant Difference post‐hoc test, using the ezANOVA software (https://people.cas.sc.edu/rorden/ezanova/index.html). Differences between groups and treatments were considered statistically significant at *p <* 0.05.

## Author Contributions

**Maria Sofia Molonia**: validation, investigation, writing – original draft preparation, visualization. **Federica Lina Salamone**: validation, formal analysis, investigation, writing – original draft preparation, visualization. **Francesco Cimino**: conceptualization, methodology, validation, resources, writing – original draft preparation, writing – review and editing, visualization, supervision, project administration. **Manuela D'Arrigo**: validation, formal analysis. **Mariateresa Cristani**: validation, formal analysis, investigation. **Luana Pulvirenti**: validation, formal analysis, investigation. **Antonella Saija**: resources, writing – review and editing. **Antonio Speciale**: conceptualization, methodology, validation, resources, writing – original draft preparation, writing – review and editing, visualization, supervision. **Edoardo Napoli**: conceptualization, methodology, validation, resources, writing – original draft preparation, writing – review and editing, supervision, project administration. All authors have read and agreed to the published version of the manuscript.

## Funding

This research was partially funded by the “Fondo di Finanziamento per le Attività Base di Ricerca FFABR Unime 2022” (granted to Antonio Speciale).

## Consent

The authors have nothing to report.

## Conflicts of Interest

The authors declare no conflicts of interest.

## Supporting information




**Supporting File 1**: cbdv70811‐sup‐0001‐SuppMat.pdf

## Data Availability

The data that support the findings of this study are available on reasonable request from the corresponding author (Francesco Cimino).
