# Supplementary material for: Phenolic Profile and In Vitro Anti‐Inflammatory Activities of Salvia officinalis L. Hydrodistillation Wastewater
Source: Chem Biodivers. 2026 Jan 8;23(1):e02271. doi: 10.1002/cbdv.202502271 (PMC12781621; doi:10.1002/cbdv.202502271)
Supplement: Supplementary file 1 — Supporting File 1: cbdv70811‐sup‐0001‐SuppMat.pdf [file CBDV-23-e02271-s001.pdf]

Supplemental material

Phenolic Profile and In Vitro Anti-Inflammatory Activities of *Salvia officinalis* L. Hydrodistillation Wastewater

Maria Sofia Molonia [1,†], Federica Lina Salamone [1,2,†], Francesco Cimino [1,*], Manuela D'Arrigo [1], Mariateresa Cristani [1], Luana Pulvirenti [2], Antonella Saija [1], Antonio Speciale [1,‡], and Edoardo Napoli [2,‡]

Materials and Methods

Sulforhodamine B (SRB) assay

The cytotoxicity effect of *Salvia officinalis* L. extract (50 – 500μM) was evaluated using the sulforhodamine B (SRB) assay after 24 hours of exposure. In summary, following treatment, Raw 264.7 and Caco-2 cells were gently washed with DPBS and fixed at 4°C for 1 hour using a 10% (w/v) trichloroacetic acid solution. Next, the fixed cells were stained with 0.4% (w/v) sulforhodamine B dissolved in 1% acetic acid for 30 minutes. Excess dye was removed by washing with 1% (v/v) acetic acid and then the protein-bound dye was solubilized in a 10 mM Tris base solution. The absorbance was measured at 565 nm using a microplate reader (GloMax® Discover System-TM397). The results are reported as the percentage (%) of viable cells relative to the untreated control group.

Total cell lysates extraction and Western Blot analysis

For Raw 264.7 cells, total protein extraction was performed by lysing cells in a buffer containing 10 mM Tris-HCl, 150 mM NaCl, 5 mM EDTA Na_2, and 0.1% (v/v) Triton. The lysates were incubated and mixed for 30 minutes at 4°C to obtain complete protein extraction, and stored at -80°C until further analysis. Protein concentration in each sample was determined using the Bradford reagent [54], with bovine serum albumin as standard. For immunoblotting analysis, 30 μg of total protein lysates was denatured and subjected to SDS-PAGE. Afterwards, proteins were transferred to PVDF membrane (Hybond-P PVDF, Amersham Bioscience). Membranes were incubated overnight at 4° C with specific primary antibodies: rabbit anti-NF-κB p65 monoclonal antibody (Cell Signaling Technology) (1:1000), and rabbit anti-β-actin monoclonal antibody (Cell Signaling Technology) (1:6000). Then, membranes were exposed to peroxidase-conjugated secondary anti-rabbit Ig (Cell Signaling Technology, Danvers, MA, USA) (1:6000) for 2 h at 4° C, and luminescence was visualized with Clarity Max System (Bio-Rad, Hercules, CA, USA). The protein loading was evaluated by

Ponceau S staining and by housekeeping protein β-actin. Quantitative measurement was performed by Image Lab software (Bio-Rad, Hercules, CA, USA).

Results

Salvia officinalis L. hydrodistillation wastewater effect on cell viability

The results showed that *Salvia officinalis* L. hydrodistillation water treatment at the concentrations chosen in the experiments (100 – 200 µg/mL for Caco-2 and 50 – 100 µg/mL for RAW 264.7 cell lines) did not exhibit significant cytotoxic effects in cell viability compared to control (CTR) (Figure S1).

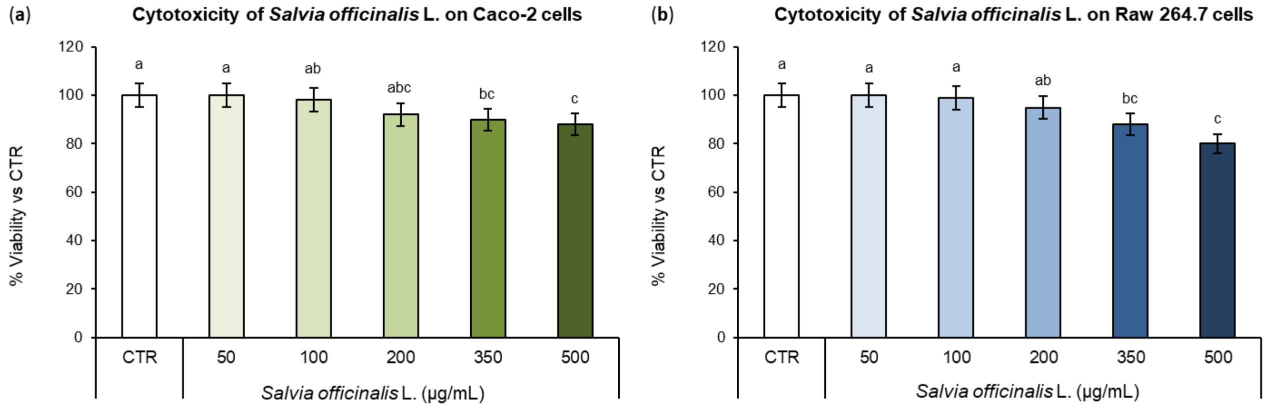

Figure S1. Cell Viability. Cytotoxicity was evaluated by SRB assay on Caco-2 (**a**) and RAW 264.7 (**b**) cells exposed to different concentrations of *Salvia officinalis* L. extract (50 – 500 µg/mL) for 24 h. Control cells were treated with the vehicle alone (DMEM full). Results are expressed as the percentage of cell viability relative to the control (CTR). All data are expressed as mean ± SD of three independent experiments (n = 3) each performed in triplicate. Means with the same letter are not significantly different from each other ($p > 0.05$).

Effect on total p65 (NF-κB) protein levels in Raw 264.7 cells

The results in Fig. 2S demonstrate that *Salvia officinalis* treatment does not alter total p65 NF-κB protein levels in Raw 264.7 cells.

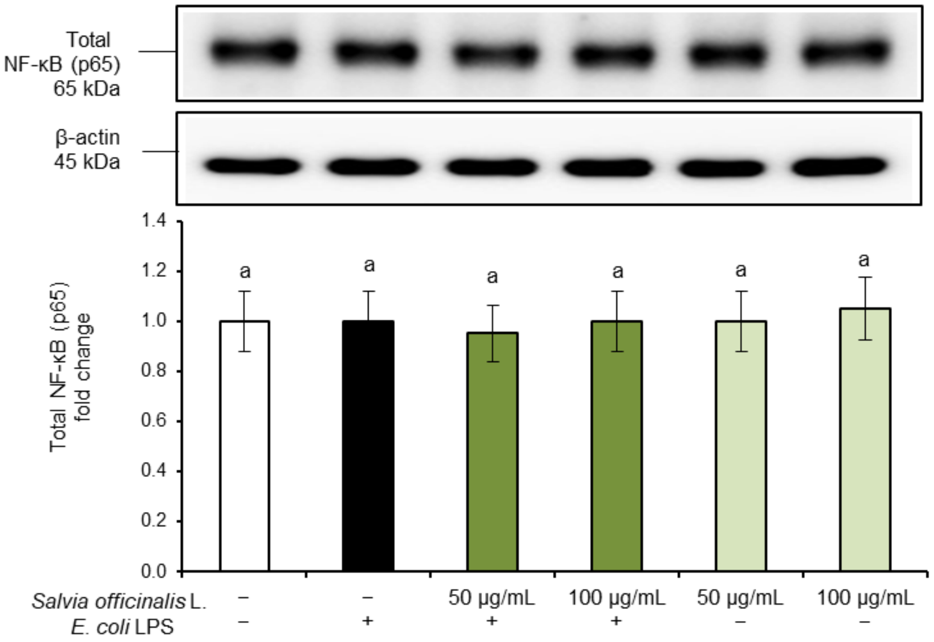

Figure S2. **Total p65 (NF-κB) protein**. Raw 264.7 cells were pre-treated for 24 hours with *Salvia officinalis* L. hydrodistillation wastewater (50 and 100 µg/mL) and then exposed to *E. coli* LPS (100 ng/mL) for 2 hours by adding it to the culture medium. Cells treated with the vehicles alone were used as controls. Total p65 (NF-κB) protein was analyzed by Western blot. The densitometry results are reported as fold change against control. Intensity values were normalized to the corresponding β-actin values. All data are expressed as mean ± SD of three independent experiments. Means with the same letter are not significantly different from each other ($p > 0.05$).
